# Reversible Conversion of Dominant Polarity in Ambipolar Polymer/Graphene Oxide Hybrids

**DOI:** 10.1038/srep09446

**Published:** 2015-03-24

**Authors:** Ye Zhou, Su-Ting Han, Prashant Sonar, Xinlei Ma, Jihua Chen, Zijian Zheng, V. A. L. Roy

**Affiliations:** 1 Department of Physics and Materials Science, City University of Hong Kong, Tat Chee Avenue, Kowloon Tong, Hong Kong SAR, China; 2 School of Chemistry, Physics and Mechanical Engineering, Queensland University of Technology (QUT), GPO Box 2434, Brisbane, QLD 4001, Australia; 3 Nanotechnology Center, Institute of Textiles and Clothing, The Hong Kong Polytechnic University, Hung Hom, Kowloon, Hong Kong SAR, China; 4 Center for Nanophase Materials Sciences, Oak Ridge National Laboratory, Oak Ridge, TN 37831, USA; 5 Shenzhen Research Institute, City University of Hong Kong, High-Tech Zone, Nanshan District, Shenzhen. 518057, China

## Abstract

The possibility to selectively modulate the charge carrier transport in semiconducting materials is extremely challenging for the development of high performance and low-power consuming logic circuits. Systematical control over the polarity (electrons and holes) in transistor based on solution processed layer by layer polymer/graphene oxide hybrid system has been demonstrated. The conversion degree of the polarity is well controlled and reversible by trapping the opposite carriers. Basically, an electron device is switched to be a hole only device or vice versa. Finally, a hybrid layer ambipolar inverter is demonstrated in which almost no leakage of opposite carrier is found. This hybrid material has wide range of applications in planar p-n junctions and logic circuits for high-throughput manufacturing of printed electronic circuits.

Over the past few years materials with balanced charge carrier transport have received special interest owing to their potential applications in light emitting transistors^1^,^2^,^3^, complementary metal oxide semiconductor (CMOS)-like logic circuits^4^,^5^,^6^, sensors^7^,^8^,^9^, spintronics^10^ and memories^11^,^12^. On this aspect, the possibility to selectively modulate the charge carrier transport in semiconducting materials is extremely attractive for the development of high performance and low-power consuming ambipolar logic circuits with low leakage currents^13^,^14^,^15^. Independent control over charge transport is also quite important for light-emitting transistors^16^. Several approaches have been developed to control the charge injection and transport such as interface modification^17^,^18^,^19^, split-gate^20^, dielectric selection^21^, exposition to oxygen^22^, introduction of buffer layers^23^,^24^, doping of other materials^25^ or thermal treatment^26^,^27^,^28^. However, most of the methods always require a systematical selection of materials and conditions to obtain a reliable result, and cannot be a collective approach for various structures. On the other hand, these approaches cannot switch the charge transport reversibly and dynamically in a controlled manner. Therefore, exploring a simple and universal technique to achieve reversible charge transport (from holes to electrons vice versa) is attractive for wide variety of applications. A balanced charge transport can be achieved by bilayer heterostructures, blended material systems or single component materials^29^,^30^,^31^,^32^,^33^,^34^. Among these strategies, polymeric semiconductors are highly promising and attractive due to their large scale solution processability^35^,^36^ and the cost-effective device fabrication procedure by using one single type of electrode^37^,^38^. To achieve ultimate success of practical polymer devices, a large number of efforts have been devoted to the synthesis of novel polymers, the development of new processing methods and the interface engineering in device architecture^39^. To achieve controlled charge transport in semiconducting polymers, introducing charge trapping sites with the following properties can be an effective approach: i) flattened surface with good interface quality with polymer; ii) large area with plenty of trapping sites; iii) easy processability with polymer to form hybrids. Graphene oxide (GO), the insulating analog of graphene, is a nonstoichiometrically oxidized sheet derived from the acid exfoliation of graphite^40^. The high density of oxygen-containing groups, planar structure with atom-thickness and excellent solution processabbility make chemically synthesized GO extremely promising as functional trapping layer in polymer bilayer hybrids.

Here we report a novel approach on systematic control over polarity (from holes to electron or vice versa) in transistors based on solution processed semiconducting polymer/GO hybrid system. Recently, diketopyrrolopyrrole (DPP) based polymers with planar conjugated bicyclic structure have shown some of the highest mobilities and got particular interest for low-cost functional electronic devices including transistors, logic circuits and photovoltaics^41^,^42^,^43^. Poly (diketopyrrolopyrrole-thiophene-benzothiadiazole-thiophene) (PDPP-TBT) is chosen in this work due to its high and balanced hole/electron mobilities^44^. The DPP and TBT blocks can improve intermolecular interactions to form π-π stacks with a large overlapping area and ordered molecular organization^45^,^46^. By inserting the solution processable GO sheets between the polymer and gate dielectric, the majority carrier type in the polymer could be dynamically switched from one type to another with applied programming gate bias. The conversion degree of the polarity is well controlled by the gate bias. To the best of our knowledge, this is the first report in which the charge transport is actively switched in a transistor in a control manner by using the polymer/GO bilayer structure. High performance ambipolar-like inverter has also been fabricated based on the hybrid transistors with controlled and reversible polarity. Our proposed architecture provides a powerful way to achieve charge conversion of one polarity to another in a controlled manner and thereby relaxing the demands of materials choice in various applications such as planar p-n junctions and ambipolar-like logic circuits.

## Results

### Polymer/GO ambipolar device structure

**Figure 1:**
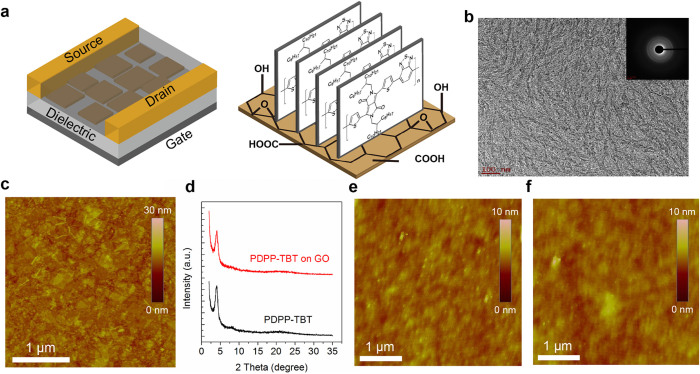
Polymer/GO based ambipolar transistor. (a) Schematic illustration of the PDPP-TBT/GO hybrids adopted in the transistor structure. (b) TEM image and SAED pattern of the polymer. Scale bar, 200 nm. (c) AFM image of the GO layer. (d) XRD patterns of the polymer and polymer/GO hybrids. Scale bar, 1 μm. (e) AFM image of the polymer on SiO_2_. Scale bar, 1 μm. (f) AFM image of the polymer on GO. Scale bar, 1 μm.

Fig. 1a show the schematic illustration of the PDPP-TBT/GO hybrids adopted in the transistor structure. GO is a layered stack of puckered sheets consisting of graphene like aromatic domains of random sizes, which are decorated by epoxy, diol, hydroxyl, ether and carboxy groups^47^,^48^. GO suspensions were prepared according to the Hummers method with pure graphite followed by exfoliation under untrasonication^49^. The atomic force microscopy (AFM) image of the synthesized GO sheet is illustrated in Supplementary Figure 1, with thickness of around 1.5 nm. Large-area uniform GO thin films were prepared by a simple spin-casting process. The degree of oxidation of graphite oxide was confirmed by XPS and the survey spectra for graphite oxide yielded C/O atomic ratios of ~2.2 as shown in Supplementary Figure 2. The Raman spectrum of the deposited GO is shown in Supplementary Figure 3. The schematic demonstration of the PDPP-TBT on GO is also shown in Fig. 1a. Transmission electron microscopy (TEM) analysis was conducted by depositing drop casted film of PDPP-TBT on carbon coated grids. TEM images and corresponding selected area electron diffraction (SAED) pattern (inset) for PDPP-TBT film annealed at 140 degree Celsius are shown in Fig. 1b. TEM images exhibit a fibrous morphology of the polymer which clearly indicates the strong intramolecular interaction. The outer ring in the SAED pattern of the film attributes to a d-spacing of 0.36 nm, which is probably due to the pi-pi stacking distance of the PDPP-TBT chains. Fig. 1c shows the tapping mode AFM image of the GO film, displaying a uniform coverage with overlaid sheets of GO. In addition, surface root-mean-square roughness (R_rms_) value of the GO film is determined to be 1.35 nm in area of 3 × 3 μm^2^. The Raman spectrum of the deposited GO is shown in Supplementary Figure 2 and the band-intensity ratio I_D_/I_G_ is about 0.96. The 30-nm-thick PDPP-TBT film was subsequently spin-casted above the GO layer and annealed at 140°C for 30 mins in nitrogen environment. To elucidate the molecular packing structure of PDPP-TBT on GO, grazing incidence X-ray diffraction (GIXRD) analysis was carried out. The crystallinity of the polymer film spin-coated on GO layer is not disturbed much as shown in Fig. 1d. The spin-cast thin film exhibits a strong primary diffraction peak at 2θ = 4.14°, corresponds to a *d*-spacing of 21.3 Å. The XRD patterns demonstrate the highly ordered lamellar packing of the polymer on GO with an edge-on orientation. The AFM images of the PDPP-TBT films grown on SiO_2_ and GO-coated SiO_2_ are shown in Fig. 1e and 1f. Both of the spin-cast thin films show uniform structures with fine grains with no obvious differences of surface morphology. Over all, the results suggest that the molecular packing of PDPP-TBT fundamentally remains unchanged when depositing on GO layer. The ambipolar transistors were fabricated on a heavily doped Si wafer (served as the gate electrode) with a thermally grown 100 nm thick SiO_2_ layer (worked as the gate dielectric). The PDPP-TBT/GO hybrids were bridged between two gold electrodes (source and drain) on top of the dielectric layer in a bottom-contact bottom-gate geometry. Further details on the device fabrication could be found at the Method section. The PDPP-TBT has a lowest unoccupied molecular orbital (LUMO) level of 4.0 eV and highest occupied molecular orbital (HOMO) level of 5.2 eV, and Au has a Fermi level in the range of 4.7–5.2 eV, therefore a low bandgap polymer is suitable as ambipolar semiconductor for hole and electron injection^44^,^50^,^51^.

### Electrical characteristics of the transistor

**Figure 2:**
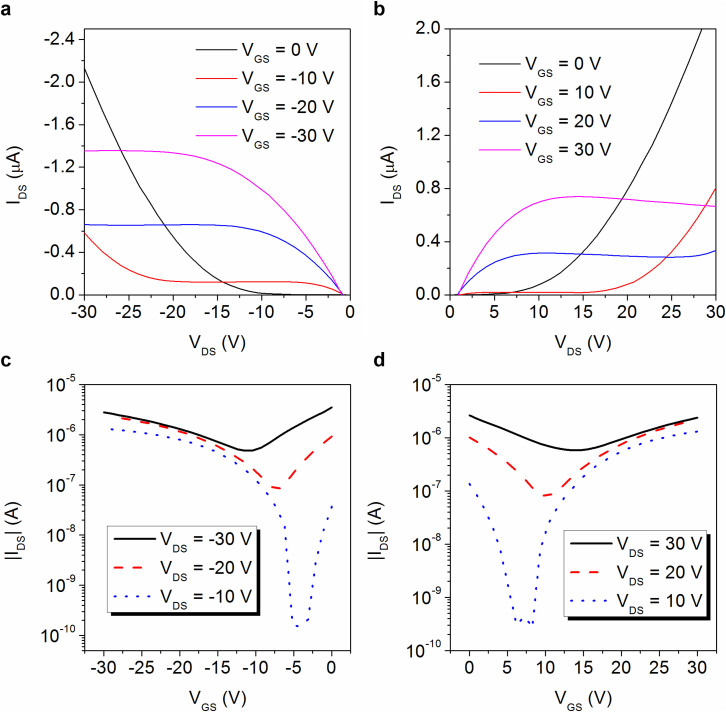
Electrical performances of the polymer/GO transistor. (a) Output characteristics of the hybrids at hole-enhancement mode. (b) Output characteristics of the hybrids at electron-enhancement mode. (c) Transfer characteristics of the hybrids at hole-enhancement mode. (d) Transfer characteristics of the hybrids at electron-enhancement mode.

The typical output characteristics of the transistors based on PDPP-TBT/GO hybrids are shown in Fig. 2a and 2b. At both high positive and negative gate–source voltage (*V*_GS_), saturation of the drain-source current (*I*_DS_) is observed, indicating electrons and holes are accumulated at the respective *V*_GS_. A slight reduction of *I*_DS_ occurred at high *V*_DS_ in electron-enhancement mode, which may be attributed to the polymer/GO interface electron trapping under large *V*_GS_. At low *V*_GS_, the transistors show diode-like curves with a rapid increase of *I*_DS_ with *V*_DS_, which is a typical behavior of ambipolar transistors due to the Schottky barrier at the junctions between source/drain to the channel^52^. Fig. 2c and 2d clearly show the V-shape transfer curves in both hole-enhancement and electron-enhancement modes. The mobility was calculated at the saturation regime using the following equation: I_DS_ = (W/2L) μC_i_(*V*_GS_ − *V*_T_)^2^, where I_DS_ is the drain-source current, W and L are the channel width and length, μ is the mobility, C_i_ is the capacitance per unit area of the gate dielectric and *V*_T_ is the threshold voltage. The hybrids exhibits a maximum hole mobility of 1.1 × 10^−2^ cm^2^ V^−1^ s^−1^ and an electron mobility of 1.2 × 10^−2^ cm^2^ V^−1^ s^−1^. To investigate the degree of charge trapping, the corresponding density of traps (N_SS_) were extracted using the equation: N_SS_ = SS log e/(kT/q − 1)(C_i_/q), where SS is the sub-threshold slope, k is the Boltzmann's constant, T is the absolute temperature and q is the fundamental unit of charge^53^. The value of SS of the hole-enhancement mode and electron-enhancement mode were 17.2 and 21.6 V/decade, respectively. The calculated values of N_SS_ were 8.8 × 10^12^ and 1.1 × 10^13^ cm^−2^, respectively.

### Polarity conversion

**Figure 3:**
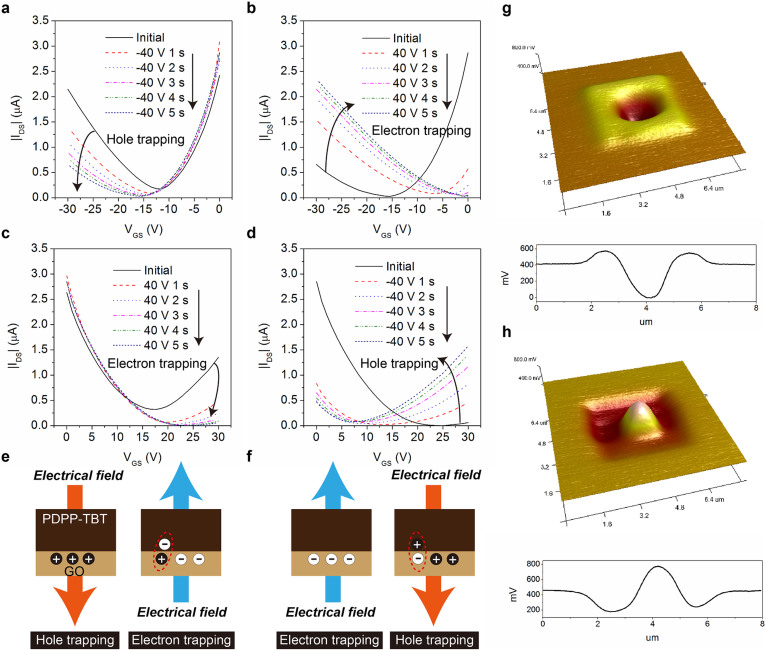
Dominant polarity conversion. (a) Transfer characteristics of the hybrids at hole-enhancement mode (V_DS_ = −30 V) after negative gate pulse. (b) Transfer characteristics of the hybrids at hole-enhancement mode (V_DS_ = −30 V) after positive gate pulse. (c) Transfer characteristics of the hybrids at electron-enhancement mode (V_DS_ = 30 V) after positive gate pulse. (d) Transfer characteristics of the hybrids at electron-enhancement mode (V_DS_ = 30 V) after negative gate pulse. (e) Schematic illustration of the hole/electron trapping process. (f) Schematic illustration of the electron/hole trapping process. (g) SKPM image of the hole/electron trapping process. (h) SKPM image of the electron/hole trapping process.

Different voltage pulses were applied to the gate to control the device polarity before the measurements. For measuring the transfer curve at the hole-enhancement mode, V_DS_ was set as −30 V and V_GS_ was swept from 0 V to −30 V. For measuring the transfer curve at the electron-enhancement mode, V_DS_ was set as 30 V and V_GS_ was swept from 0 V to 30 V. The comparison between the transfer curves, as shown in Fig. 3a to 3d, provides a clear evidence of control over ambipolar (superimposition of both electrons and holes) properties of the transistors by the pre-applied gate bias. Fig. 3a shows the transfer characteristics at hole-enhancement mode after applying −40 V at the gate for various durations. The negatively shifted switch-on voltages indicate that more and more holes are trapped in the process and the potential evolves from these traps screens the gate voltage. When a positive (+40 V) bias is pre-applied to the gate and the switch-on voltage shifted towards the positive direction as shown in Fig. 3b. Due to the positive bias, the stored holes are neutralized by electrons and eventually electrons are trapped in the GO layer. The origin of charge carrier trapping in the hybrid materials is attributed to the inherent structural defects in chemically synthesized GO and the insulating gap of oxidized GO domains originated from the oxygenated functional group^54^,^55^,^56^. The ambipolar charge transport is controlled by the amount and polarity of trapped charges by GO. The trapped charge carriers in GO can screen the gate voltage and the switch-on voltage can be manipulated according to the polarity of the trapped charge carriers. The major charge carriers during operation can be determined with controlled switch-on voltage. A pre-applied −40 V gate pulse can induce an electron-rich device, and a pre-applied +40 V gate pulse can induce a hole-rich device. The schematic illustration of the mechanism is shown in Supplementary Figure 4. The transfer characteristics at the electron-enhancement mode have also been investigated as shown in Fig. 3c and 3d. The gate was biased by various durations of +40 V to trap the electrons at the GO layer. After the application of −40 V at the gate, the holes are hopping from the polymer to GO in order to compensate the trapped electrons. Further increase of gate bias time results in hole trapping and the transfer curves shift towards more negative direction. With selective voltage pulse programmed at the gate electrode, dominant p-type or n-type transport can be achieved in the ambipolar hybrid transistors. The device without GO was also fabricated as a control device and did not exhibit controlled transport using the same approach and the results are shown in Supplementary Figure 5. In order to further confirm this approach in general to any ambipolar polymer, a hybrid transistor using poly{3,6-difuran-2-yl-2,5-di(2-octyldodecyl)-pyrrolo[3,4-*c*]pyrrole-1,4-dione-alt-benzothidiazole} (PDPP-FBF)^57^ based on the same device structure has been fabricated as shown in Supplementary Figure 6. The electrical characteristics of the ambipolar transistor are included in Supplementary Figure 7, and the hybrid transistor exhibits reliable and reversible conversion of the major charge carrier transport. The results shown here clearly demonstrate that the GO based bilayer hybrid structure is an efficient tool to manipulate the charge transport in an ambipolar semiconductor.

The working mechanism is schematically shown in Fig. 3e and 3f. The density of trapped holes and electrons in the conversion process is estimated from the equation Q = CΔ*V*_th_, where C is the capacitance per unit area and ΔV_th_ is the threshold voltage shift. The Δ*V*_th_ was 21.6 V for the electron-enhancement mode and 17.1 V for the hole-enhancement mode. The total density of traps density was found to be 4.7 × 10^12^ cm^−2^ for the electron-enhancement mode and 3.7 × 10^12^ cm^−2^ for the hole-enhancement mode. The high density of charge traps indicates that large amounts of oxygen trap centers exit in the chemically synthesized GO thin films, which lead to efficient charge trapping and de-trapping of the hybrid films. In order to investigate the charge trapping ability of GO, scanning Kelvin probe microscopy (SKPM) was used for real-space imaging of hole and electron trapping states. Charges stored within the GO are detected from the changes in the surface potential and the detailed measuring methods can be found at the experimental section. The charge injection operation was first performed within an area of 4 × 4 μm^2^ with +6 V bias applied at the tip and holes were confined in the GO. In the following process, the center 1 × 1 μm^2^ was scanned by applying a −6 V bias. During this process, the originally trapped holes recombine with electrons and electron trapping occurs. Fig. 3g shows a typical real-space SKPM image of hole trapping states followed by electron trapping states. The yellow region identifies hole trapping state and dark region corresponds to electron trapping state. The potential difference between the two states was measured to be approximately 575 mV. The SKPM image of electron trapping states followed by hole trapping states is shown in Fig. 3h. A −6 V bias at the tip trapped the electrons within an area of 4 × 4 μm^2^ followed by the scanning of the center 1 × 1 μm^2^ with a +6 V bias to form hole trapping states. The potential difference between the two states was measured to be about 590 mV. These results show that GO can act as efficient hole/electron trapping elements and enables a wide range of traps in our device. The trapped charge carriers can remain inside the GO for more than 1 hour after the applied bias has been removed, resulting in a stable conversion of dominant polarity in the hybrid transistors.

### Ambipolar inverter

**Figure 4:**
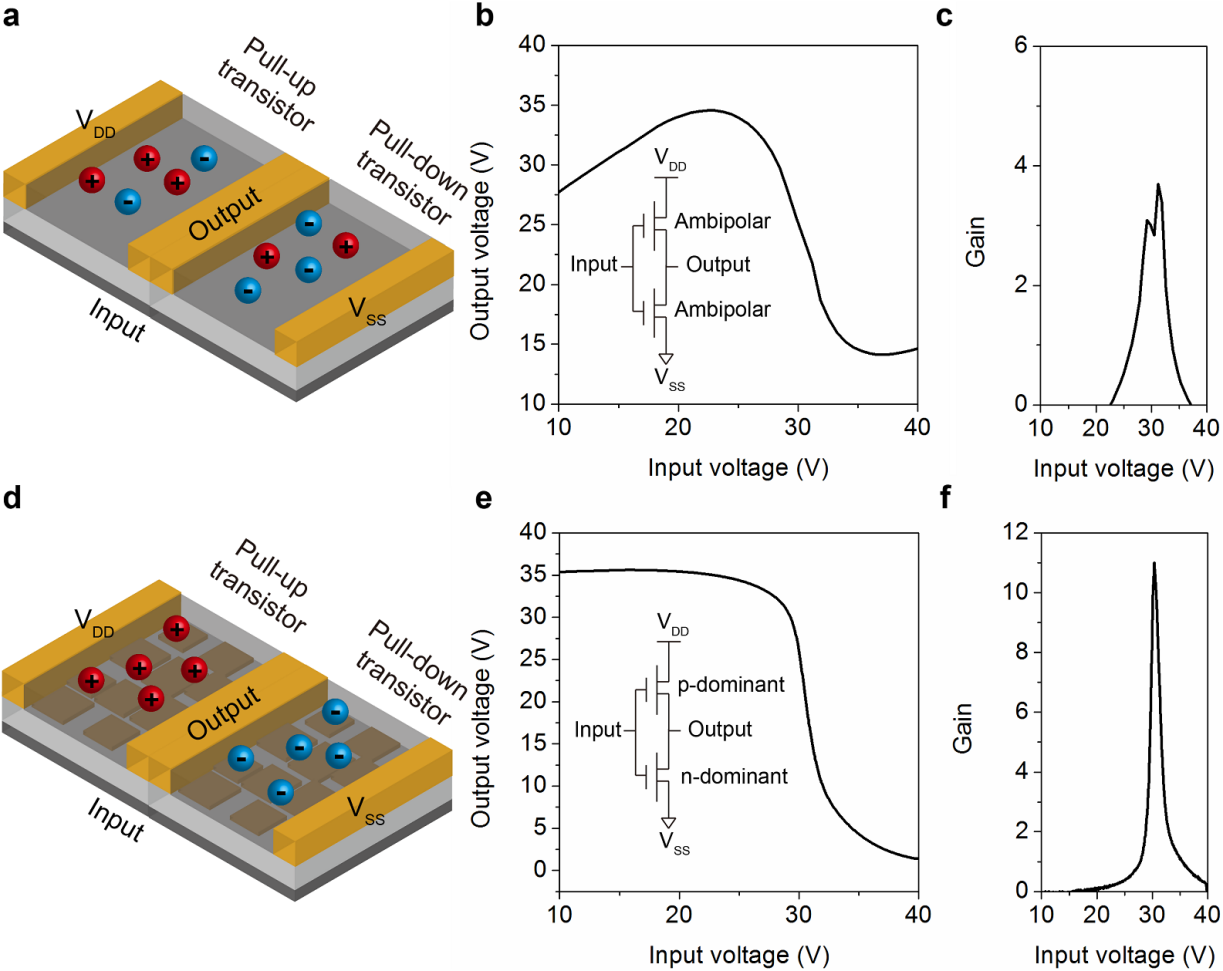
Ambipolar inverters. (a) Schematic illustration of the PDPP-TBT based inverter. (b) Voltage transfer curve of the PDPP-TBT based inverter. (c) Signal gain of the PDPP-TBT based inverter. (d) Schematic illustration of the PDPP-TBT/GO based inverter. (e) Voltage transfer curve of the PDPP-TBT/GO based inverter. (f) Signal gain of the PDPP-TBT/GO based inverter.

In order to demonstrate the application of dominant polarity conversion in logic circuits, we constructed an ambipolar inverter based on the ambipolar transistors. Two identical ambipolar transistors are combined with a common gate as the input and a common drain as the output as shown in Fig. 4a. The voltage transfer characteristic (VTC) of the inverter with only PDPP-TBT is shown in Fig. 4b and the signal gain (−d*V*_OUT_/d*V*_IN_) is shown in Fig. 4c. Typical Z-shaped VTC was observed in the PDPP-TBT inverter, since both the pull-down (n-channel) transistor and pull-up (p-channel) transistor could not be switched off at low and high input voltage values. This will result in high static power consumption and limited noise margins compared with conventional complementary logic circuits^58^,^59^. Therefore, it is crucial to achieve unipolar operation in the p-channel and n-channel ambipolar transistors. We further applied the PDPP-TBT/GO hybrid transistors in the inverter structure as illustrated in Fig. 4d. The p-channel transistor was programmed with +40 V at gate for 10 s and the n-channel transistor was programmed with −40 V at gate for 10 s. The VTC and signal gain of the hybrid inverter are shown in Fig. 4e and 4f. The inverter exhibits clearly an improved static performance with large signal gain and sharp switching with rail-to-rail output swings, which is comparable with other reported inverters^60^,^61^. The realization of dominant p-type transport in pull-up transistor and dominant n-type transport in pull-down transistor play an important role in fabricating high performance ambipolar inverters. Further improvement of the ambipolar inverter's performance can be realized by systematical tuning of the pull-up and pull-down transistor with matched switch-on voltage.

## Discussion

We have demonstrated an innovative way to manipulate the charge transport properties of ambipolar polymers by GO. The GO influences the majority carrier transport in the transistor operation when acting as electron/hole trapping site. The majority carrier type in the ambipolar polymer could be dynamically switched from one type to another with applied programming gate bias. The device works in a charge trapping mechanism and the electron/hole trapping ability has been confirmed by SKPM. High performance ambipolar inverter has been fabricated from the PDPP-TBT/GO hybrid transistors with well controlled dominant polarity. We believe that this method offers a versatile opportunity to achieve conversion of polarity in a controlled manner. The ambipolar polymer/GO hybrids would become potentially useful for high-throughput manufacturing of printed circuits for a wide range of electronic applications.

## Methods

### Materials

All chemicals were obtained from Aldrich and used without further purification. Detailed synthetic procedures for the preparation of PDPP-TBT and PDPP-FBF have been published previously^44^,^57^.

### Fabrication of ambipolar transistors

A highly doped silicon wafer with a thermally grown 100-nm-thick SiO_2_ layer was utilized as the substrate for transistors. The 100-nm-thick gold source/drain electrodes were patterned by photolithography. The active channel length and width were 10 μm and 3000 μm, respectively. The wafers were subjected to cleaning using ultrasonication in acetone, iso-propanol and de-ionized water. The substrates were then dried under a nitrogen flow and treated in UV-ozone for 15 min before use. GO was synthesized by the Hummers method and exfoliated under ultrasonication to yield a brown dispersion of GO in water. The GO thin film was deposited on the cleaned substrate by spin-casting at 3000 rpm for 30 s from the prepared GO suspension (1 mg/ml). Next, the substrates were transferred into a nitrogen glove box for all subsequent processing steps. The PDPP-TBT was spin-casted from chloroform (7 mg/ml) on GO and then annealed at 140°C for 30 min. The PDPP-FBF was spin-casted from the polymer solution in chloroform (8 mg/ml) and subsequently annealed at 140°C for 30 min.

### Characterization

The electrical characteristics were recorded in a nitrogen-filled glove box by using a Keithley 2612 source meter and 2400 source meter. Surface morphologies of the deposited films were studied using an atomic force microscope (AFM, Veeco Multimode V) in the tapping mode. The grazing incidence X-ray diffraction (GIXRD) patterns of PDPP-TBT films were recorded using an X-ray diffractometer (Rigaku SmartLab). Irradiation of parallel CuK_α1,2_ X-ray beam was fixed at a grazing incident angle of 0.5° and the detector was independently moved to collect the diffraction data with a step-size of 0.02°. The SKPM was performed in the AFM system (Bruker NanoScope 8) with metalized probes based on previous work^62^. Heavily n-doped Si wafers were used as the substrates and the GO were sandwiched between 30 nm thick Al_2_O_3_ and 10 nm thick PMMA. Al_2_O_3_ were deposited using a Savannah 100 atomic layer deposition (ALD) system at a substrate temperature of 80°C. PMMA films were spin-casted on top of GO from a solution containing 3 mg/ml PMMA in toluene and then annealed at 120°C for 1 hr. Potential images were realized under the lift-mode with a pre-defined lift height of 50 nm.

## Author Contributions

Y.Z. and S.T.H. performed the experiments and wrote the paper. P.S. synthesized the polymer. X.M. and Z.Z. assisted with kelvin probe measurements. J.C. did the TEM measurements. V.A.L.R. acquires the idea, supervised the project and finalized the manuscript.

## Supplementary Material

Supplementary Information Supplementary Dataset

## References

[b1] Zaumseil J., Friend R. H. & Sirringhaus H. Spatial control of the recombination zone in an ambipolar light-emitting organic transistor. Nat. Mater. 5, 69–74 (2006).

[b2] Capelli R. *et al.* Organic light-emitting transistors with an efficiency that outperforms the equivalent light-emitting diodes. Nat. Mater. 9, 496–503 (2010). 20436466 10.1038/nmat2751

[b3] Bürgi L. *et al.* High-Mobility Ambipolar Near-Infrared Light-Emitting Polymer Field-Effect Transistors. Adv. Mater. 20, 2217–2224 (2008).

[b4] Smith J. *et al.* Air-Stable Solution-Processed Hybrid Transistors with Hole and Electron Mobilities Exceeding 2 cm2 V−1 s−1. Adv. Mater. 22, 3598–3602 (2010). 20665561 10.1002/adma.201000195

[b5] Eda G., Fanchini G. & Chhowalla M. Large-area ultrathin films of reduced graphene oxide as a transparent and flexible electronic material. Nat. Nanotechnol. 3, 270–274 (2008). 18654522 10.1038/nnano.2008.83

[b6] Li L. *et al.* Black phosphorus field-effect transistors. Nat. Nanotechnol. 9, 372–377 (2014). 24584274 10.1038/nnano.2014.35

[b7] Traversi F. *et al.* Detecting the translocation of DNA through a nanopore using graphene nanoribbons. Nat. Nanotechnol. 8, 939–945 (2013). 24240429 10.1038/nnano.2013.240

[b8] Ji S., Wang H., Wang T. & Yan D. A High-Performance Room-Temperature NO2 Sensor Based on An Ultrathin Heterojunction Film. Adv. Mater. 25, 1755–1760 (2013). 23381844 10.1002/adma.201204134

[b9] Park J.-U., Nam S., Lee M.-S. & Lieber C. M. Synthesis of monolithic graphene–graphite integrated electronics. Nat. Mater. 11, 120–125 (2012). 22101813 10.1038/nmat3169 PMC3602909

[b10] Kong D. *et al.* Ambipolar field effect in the ternary topological insulator (BixSb1-x)2Te3 by composition tuning. Nat. Nanotechnol. 6, 705–709 (2011). 21963714 10.1038/nnano.2011.172

[b11] Sup Choi M. *et al.* Controlled charge trapping by molybdenum disulphide and graphene in ultrathin heterostructured memory devices. Nat. Commun. 4, 1624 (2013). 23535645 10.1038/ncomms2652

[b12] Zhou Y., Han S.-T., Sonar P. & Roy V. A. L. Nonvolatile multilevel data storage memory device from controlled ambipolar charge trapping mechanism. Sci. Rep. 3, 2319 (2013). 23900459 10.1038/srep02319 PMC3728587

[b13] Ribierre J.-C. *et al.* Reversible Conversion of the Majority Carrier Type in Solution-Processed Ambipolar Quinoidal Oligothiophene Thin Films. Adv. Mater. 22, 4044–4048 (2010). 20677190 10.1002/adma.201001170

[b14] Yan H. *et al.* Programmable nanowire circuits for nanoprocessors. Nature 470, 240–244 (2011). 21307937 10.1038/nature09749

[b15] Klauk H., Zschieschang U., Pflaum J. & Halik M. Ultralow-power organic complementary circuits. Nature 445, 745–748 (2007). 17301788 10.1038/nature05533

[b16] Roelofs W. S. C., Adriaans W. H., Janssen R. A. J., Kemerink M. & de Leeuw D. M. Light Emission in the Unipolar Regime of Ambipolar Organic Field-Effect Transistors. Adv. Funct. Mater. 23, 4133–4139 (2013).

[b17] Benson N., Schidleja M., Melzer C., Schmechel R. & von Seggern H. Complementary organic field effect transistors by ultraviolet dielectric interface modification. Appl. Phys. Lett. 89, 182105 (2006).

[b18] Cheng X. *et al.* Controlling Electron and Hole Charge Injection in Ambipolar Organic Field-Effect Transistors by Self-Assembled Monolayers. Adv. Funct. Mater. 19, 2407–2415 (2009).

[b19] Chen W., Qi D.-C., Huang H., Gao X. & Wee A. T. S. Organic–Organic Heterojunction Interfaces: Effect of Molecular Orientation. Adv. Funct. Mater. 21, 410–424 (2011).

[b20] Hsu B. B. Y. *et al.* Split-Gate Organic Field Effect Transistors: Control Over Charge Injection and Transport. Adv. Mater. 22, 4649–4653 (2010). 20839245 10.1002/adma.201001509

[b21] Saudari S. R., Lin Y. J., Lai Y. & Kagan C. R. Device Configurations for Ambipolar Transport in Flexible, Pentacene Transistors. Adv. Mater. 22, 5063–5068 (2010). 20941773 10.1002/adma.201001853

[b22] Tada H., Touda H., Takada M. & Matsushige K. Quasi-intrinsic semiconducting state of titanyl-phthalocyanine films obtained under ultrahigh vacuum conditions. Appl. Phys. Lett. 76, 873–875 (2000).

[b23] Takashima W., Murasaki T., Nagamatsu S., Morita T. & Kaneto K. Unipolarization of ambipolar organic field effect transistors toward high-impedance complementary metal-oxide-semiconductor circuits. Appl. Phys. Lett. 91, 071905 (2007).

[b24] Zhang Y. *et al.* DNA Interlayers Enhance Charge Injection in Organic Field-Effect Transistors. Adv. Mater. 24, 4255–4260 (2012). 22718359 10.1002/adma.201201248

[b25] El Gemayel M. *et al.* Leveraging the Ambipolar Transport in Polymeric Field-Effect Transistors via Blending with Liquid-Phase Exfoliated Graphene. Adv. Mater. 26, 4814–4819 (2014). 24862253 10.1002/adma.201400895

[b26] Tsao H. N. *et al.* From Ambi- to Unipolar Behavior in Discotic Dye Field-Effect Transistors. Adv. Mater. 20, 2715–2719 (2008). 25213895 10.1002/adma.200702992

[b27] Lee J. *et al.* Inversion of Dominant Polarity in Ambipolar Polydiketopyrrolopyrrole with Thermally Removable Groups. Adv. Funct. Mater. 22, 4128–4138 (2012).

[b28] Huettner S. *et al.* Tunable Charge Transport Using Supramolecular Self-Assembly of Nanostructured Crystalline Block Copolymers. ACS Nano 5, 3506–3515 (2011). 21500861 10.1021/nn200647d

[b29] Dodabalapur A., Katz H. E., Torsi L. & Haddon R. C. Organic Heterostructure Field-Èffect Transistors. Science 269, 1560–1562 (1995). 17789448 10.1126/science.269.5230.1560

[b30] Meijer E. J. *et al.* Solution-processed ambipolar organic field-effect transistors and inverters. Nat. Mater. 2, 678–682 (2003). 14502272 10.1038/nmat978

[b31] Zhou Y., Han S.-T., Xu Z.-X. & Roy V. A. L. Controlled Ambipolar Charge Transport Through a Self-Assembled Gold Nanoparticle Monolayer. Adv. Mater. 24, 1247–1251 (2012). 22298461 10.1002/adma.201104375

[b32] Huang X. *et al.* Graphene-Based Materials: Synthesis, Characterization, Properties, and Applications. Small 7, 1876–1902 (2011). 21630440 10.1002/smll.201002009

[b33] Zhao Y., Guo Y. & Liu Y. 25th Anniversary Article: Recent Advances in n-Type and Ambipolar Organic Field-Effect Transistors. Adv. Mater. 25, 5372–5391 (2013). 24038388 10.1002/adma.201302315

[b34] Bisri S. Z., Piliego C., Gao J. & Loi M. A. Outlook and Emerging Semiconducting Materials for Ambipolar Transistors. Adv. Mater. 26, 1176–1199 (2014). 24591008 10.1002/adma.201304280

[b35] Lu G. *et al.* Moderate doping leads to high performance of semiconductor/insulator polymer blend transistors. Nat. Commun. 4, 1588 (2013). 23481396 10.1038/ncomms2587

[b36] Knopfmacher O. *et al.* Highly stable organic polymer field-effect transistor sensor for selective detection in the marine environment. Nat. Commun. 5, 2954 (2014). 24389531 10.1038/ncomms3954

[b37] Zaumseil J. & Sirringhaus H. Electron and Ambipolar Transport in Organic Field-Effect Transistors. Chem. Rev. 107, 1296–1323 (2007). 17378616 10.1021/cr0501543

[b38] Yuen J. D. *et al.* High Performance Weak Donor–Acceptor Polymers in Thin Film Transistors: Effect of the Acceptor on Electronic Properties, Ambipolar Conductivity, Mobility, and Thermal Stability. J. Am. Chem. Soc. 133, 20799–20807 (2011). 22043809 10.1021/ja205566w

[b39] Baeg K.-J., Caironi M. & Noh Y.-Y. Toward Printed Integrated Circuits based on Unipolar or Ambipolar Polymer Semiconductors. Adv. Mater. 25, 4210–4244 (2013). 23761043 10.1002/adma.201205361

[b40] Zhu Y. *et al.* Graphene and Graphene Oxide: Synthesis, Properties, and Applications. Adv. Mater. 22, 3906–3924 (2010). 20706983 10.1002/adma.201001068

[b41] Nielsen C. B., Turbiez M. & McCulloch I. Recent Advances in the Development of Semiconducting DPP-Containing Polymers for Transistor Applications. Adv. Mater. 25, 1859–1880 (2013). 23008141 10.1002/adma.201201795

[b42] Li Y. *et al.* Annealing-Free High-Mobility Diketopyrrolopyrrole−Quaterthiophene Copolymer for Solution-Processed Organic Thin Film Transistors. J. Am. Chem. Soc. 133, 2198–2204 (2011). 21271705 10.1021/ja1085996

[b43] Li Y., Sonar P., Murphy L. & Hong W. High mobility diketopyrrolopyrrole (DPP)-based organic semiconductor materials for organic thin film transistors and photovoltaics. Energy & Environmental Science 6, 1684–1710 (2013).

[b44] Sonar P., Singh S. P., Li Y., Soh M. S. & Dodabalapur A. A Low-Bandgap Diketopyrrolopyrrole-Benzothiadiazole-Based Copolymer for High-Mobility Ambipolar Organic Thin-Film Transistors. Adv. Mater. 22, 5409–5413 (2010). 20945426 10.1002/adma.201002973

[b45] McCulloch I. *et al.* Liquid-crystalline semiconducting polymers with high charge-carrier mobility. Nat. Mater. 5, 328–333 (2006). 16547518 10.1038/nmat1612

[b46] Pan H. *et al.* Low-Temperature, Solution-Processed, High-Mobility Polymer Semiconductors for Thin-Film Transistors. J. Am. Chem. Soc. 129, 4112–4113 (2007). 17362006 10.1021/ja067879o

[b47] Jeong H.-K. *et al.* Evidence of Graphitic AB Stacking Order of Graphite Oxides. J. Am. Chem. Soc. 130, 1362–1366 (2008). 18179214 10.1021/ja076473o

[b48] Zhuang X.-D. *et al.* Conjugated-Polymer-Functionalized Graphene Oxide: Synthesis and Nonvolatile Rewritable Memory Effect. Adv. Mater. 22, 1731–1735 (2010). 20496405 10.1002/adma.200903469

[b49] Hummers W. S. & Offeman R. E. Preparation of Graphitic Oxide. J. Am. Chem. Soc. 80, 1339–1339 (1958).

[b50] Braun S., Salaneck W. R. & Fahlman M. Energy-Level Alignment at Organic/Metal and Organic/Organic Interfaces. Adv. Mater. 21, 1450–1472 (2009).

[b51] Wang H. & Donghang Y. Organic heterostructures in organic field-effect transistors. NPG Asia Mater. 2, 69–78 (2010).

[b52] Yoon M.-H., DiBenedetto S. A., Facchetti A. & Marks T. J. Organic Thin-Film Transistors Based on Carbonyl-Functionalized Quaterthiophenes: High Mobility N-Channel Semiconductors and Ambipolar Transport. J. Am. Chem. Soc. 127, 1348–1349 (2005). 15686347 10.1021/ja045124g

[b53] Cheng S.-S. *et al.* Solution-Processed Small-Molecule Bulk Heterojunction Ambipolar Transistors. Adv. Funct. Mater. 24, 2057–2063 (2014).

[b54] Chen J.-H., Cullen W. G., Jang C., Fuhrer M. S. & Williams E. D. Defect Scattering in Graphene. Phys. Rev. Lett. 102, 236805 (2009). 19658959 10.1103/PhysRevLett.102.236805

[b55] Wang S., Pu J., Chan D. S. H., Cho B. J. & Loh K. P. Wide memory window in graphene oxide charge storage nodes. Appl. Phys. Lett. 96, 143109 (2010).

[b56] Kim T.-W. *et al.* Graphene oxide nanosheets based organic field effect transistor for nonvolatile memory applications. Appl. Phys. Lett. 97, 023310 (2010).

[b57] Sonar P., Foong T. R. B., Singh S. P., Li Y. & Dodabalapur A. A furan-containing conjugated polymer for high mobility ambipolar organic thin film transistors. Chem. Commun. 48, 8383–8385 (2012). 10.1039/c2cc33093h 22798995

[b58] Chen H., Cao Y., Zhang J. & Zhou C. Large-scale complementary macroelectronics using hybrid integration of carbon nanotubes and IGZO thin-film transistors. Nat. Commun. 5, 4097 (2014). 24923382 10.1038/ncomms5097

[b59] Ding L. *et al.* CMOS-based carbon nanotube pass-transistor logic integrated circuits. Nat. Commun. 3, 677 (2012). 22334080 10.1038/ncomms1682 PMC3293427

[b60] Yu W. J. *et al.* Vertically stacked multi-heterostructures of layered materials for logic transistors and complementary inverters. Nat. Mater. 12, 246–252 (2013). 23241535 10.1038/nmat3518 PMC4249642

[b61] Fukuda K. *et al.* Fully-printed high-performance organic thin-film transistors and circuitry on one-micron-thick polymer films. Nat. Commun. 5, 4147 (2014). 24948030 10.1038/ncomms5147

[b62] Ma X. *et al.* Polymer Brush Electrets. Adv. Funct. Mater. 23, 3239–3246 (2013).

