# Supplementary material for: Reversible Conversion of Dominant Polarity in Ambipolar Polymer/Graphene Oxide Hybrids
Source: Sci Rep. 2015 Mar 24;5:9446. doi: 10.1038/srep09446 (PMC4371103; doi:10.1038/srep09446)
Supplement: Supplementary Information — Supplementary Dataset [file srep09446-s1.doc]

Supplementary Information

Reversible Conversion of Dominant Polarity in Ambipolar Polymer/Graphene Oxide Hybrids

Ye Zhou,^1^ Su-Ting Han,^1^ Prashant Sonar,^2^ Xinlei Ma,^3^ Jihua Chen,^4^ Zijian Zheng,^3^ and V. A. L. Roy^1,5*^

^1^Department of Physics and Materials Science, Tat Chee Avenue, Kowloon Tong, City University of Hong Kong, Hong Kong SAR, China

^2^School of Chemistry, Physics and Mechanical Engineering, Queensland University of Technology (QUT), GPO Box 2434, Brisbane, QLD 4001, Australia

^3^Nanotechnology Center, Institute of Textiles and Clothing, The Hong Kong Polytechnic University, Hung Hom, Kowloon, Hong Kong SAR, China

^4^Center for Nanophase Materials Sciences, Oak Ridge National Laboratory, Oak Ridge, TN 37831, USA

^5^Shenzhen Research Institute, City University of Hong Kong, High-Tech Zone, Nanshan District, Shenzhen, 518057, China


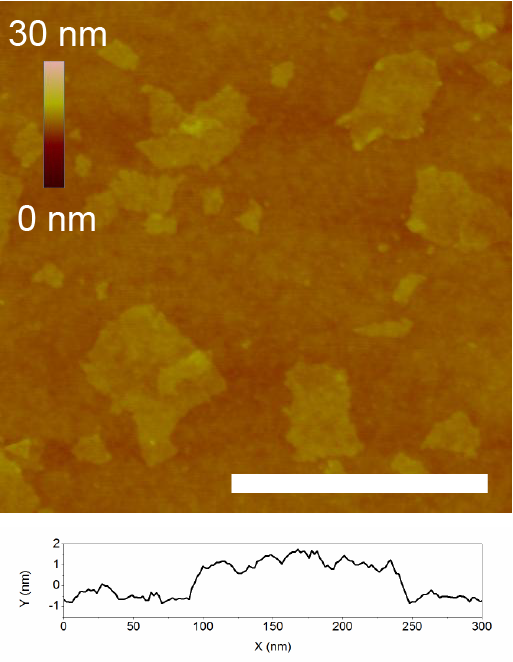


**Supplementary Figure 1.** AFM image of the synthesized GO sheet and corresponding height profile. Scale bar, 500 nm.


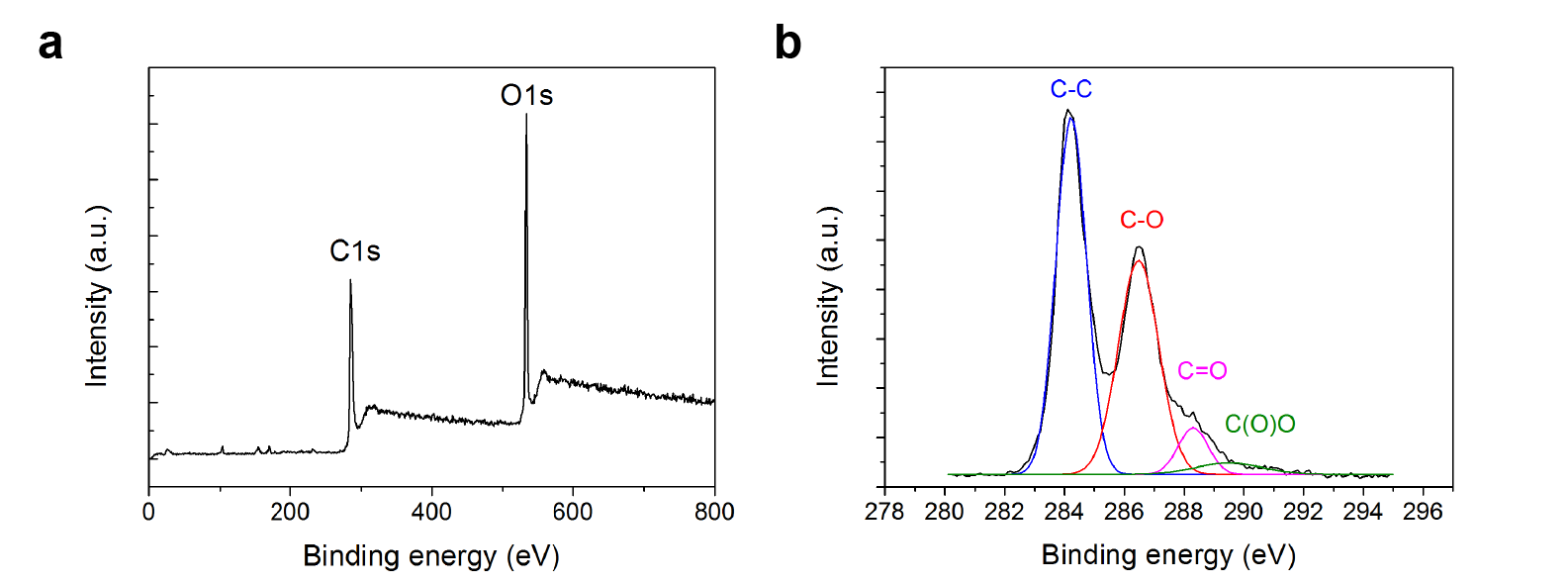


**Supplementary Figure 2.** (a) The survey spectra for graphite oxide. (b) C1s signal of GO obtained by XPS can be fitted by four components: C-C, C-O, C=O and O=C-O.


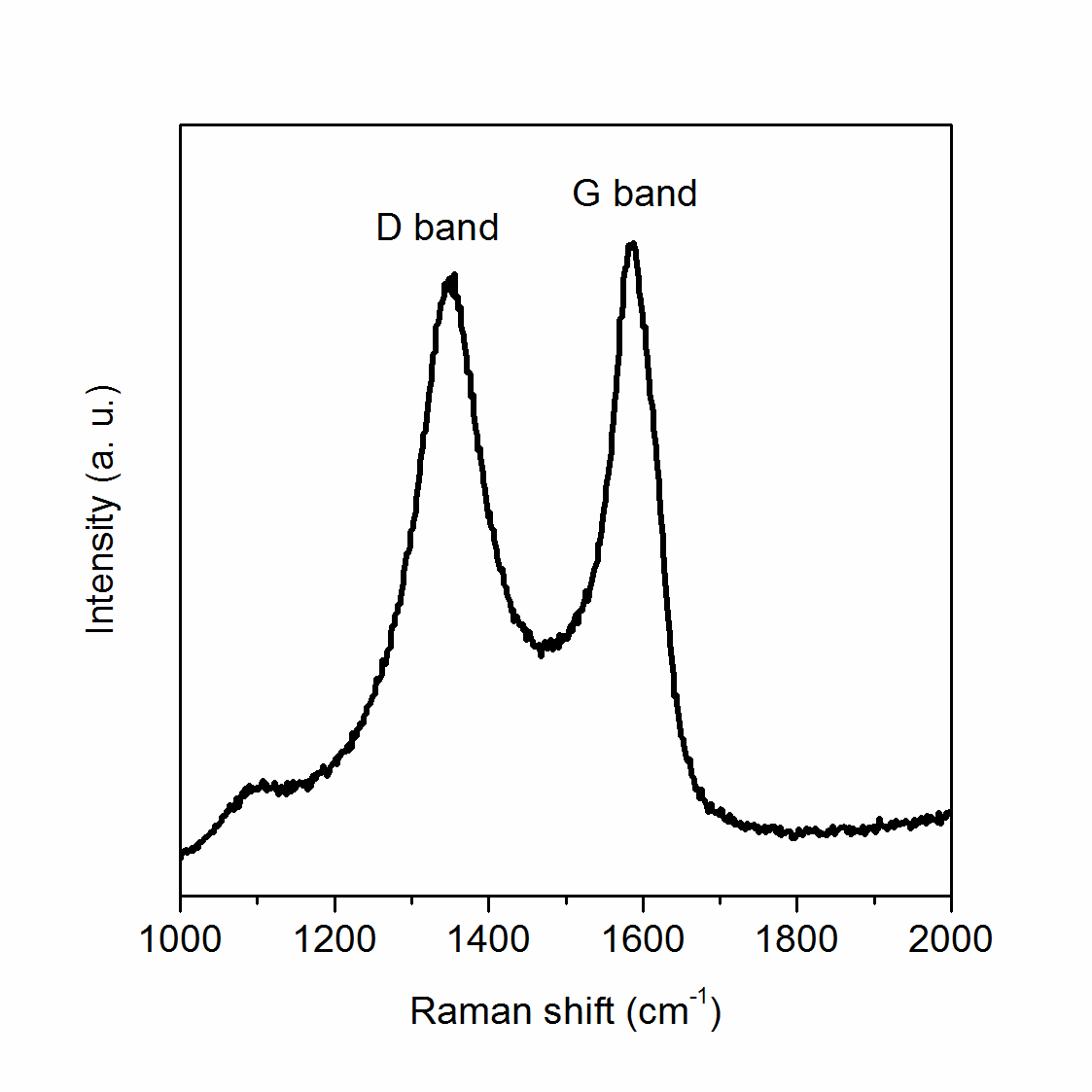


**Supplementary Figure 3.** The Raman spectrum of the deposited GO.


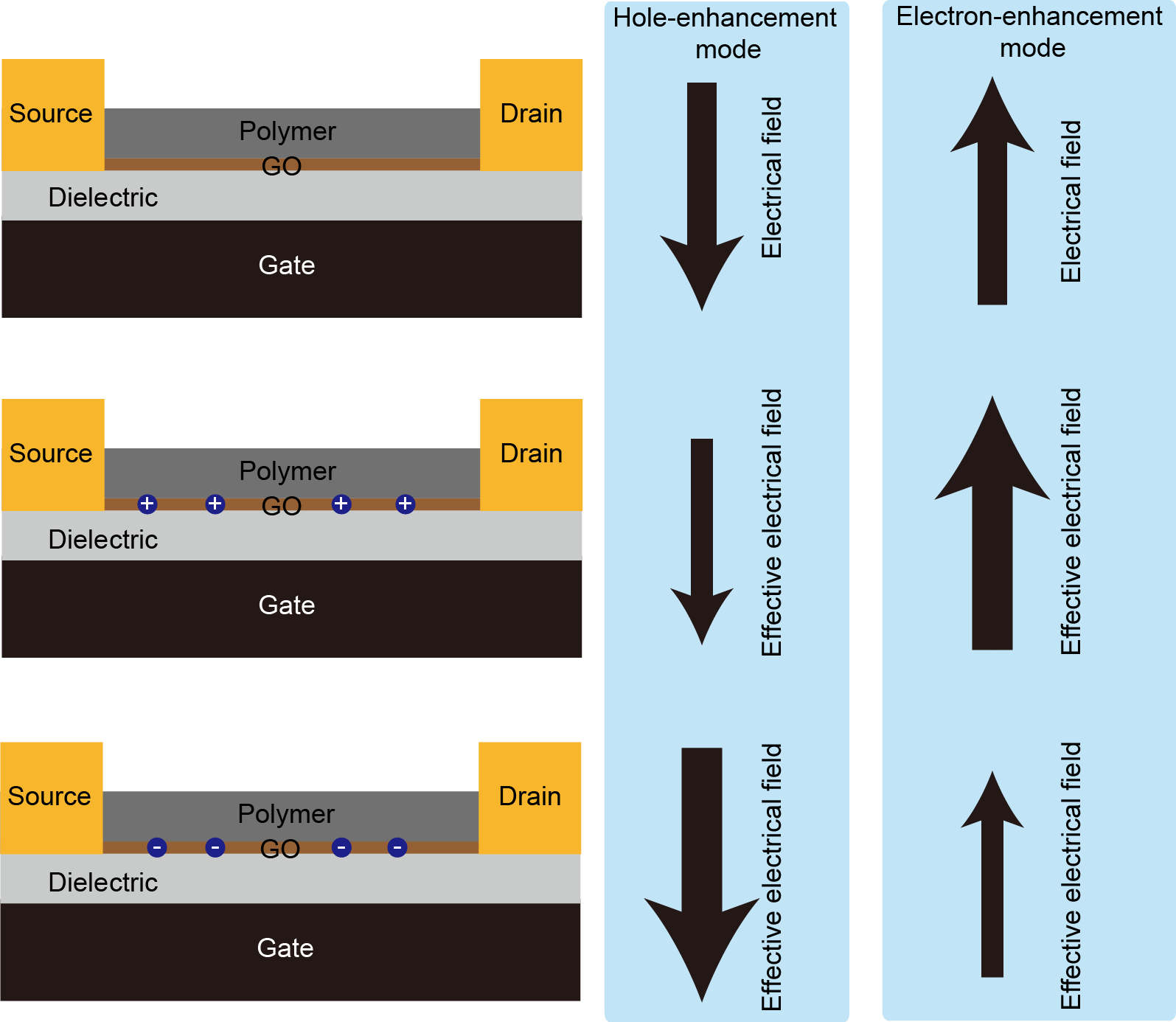


**Supplementary Figure 4.** The proposed mechanism of the controlled ambipolar transistor. The top figure shows that no holes/electrons are trapped when there is no pre-applied gate pluse. The electrical fields at hole-enhancement mode and electron-enhancement mode are shown as arrows. The middle figure shows that holes are trapped in GO after the pre-applied negative gate pulse. The effective electrical field decreases at hole-enhancement mode and increases at electron-enhancement mode. The bottom figure shows that electrons are trapped in GO after the pre-applied positive gate pulse. The effective electrical field increases at hole-enhancement mode and decreases at electron-enhancement mode.


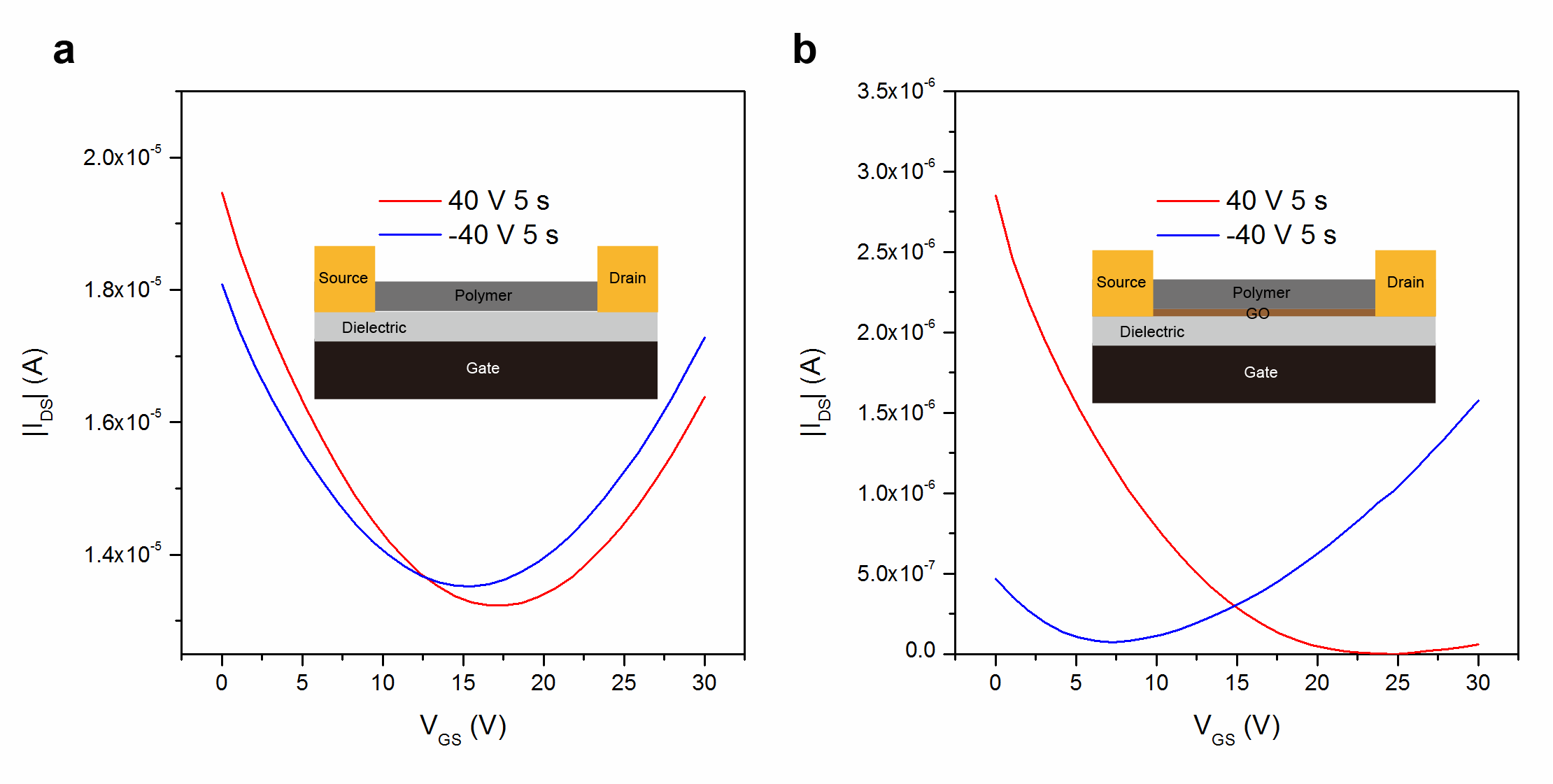


**Supplementary Figure 5.** (a) Transfer characteristic of the PDPP-TBT transistor after applying the gate pulse. (b) Transfer characteristic of the PDPP-TBT/GO transistor after applying the gate pulse.


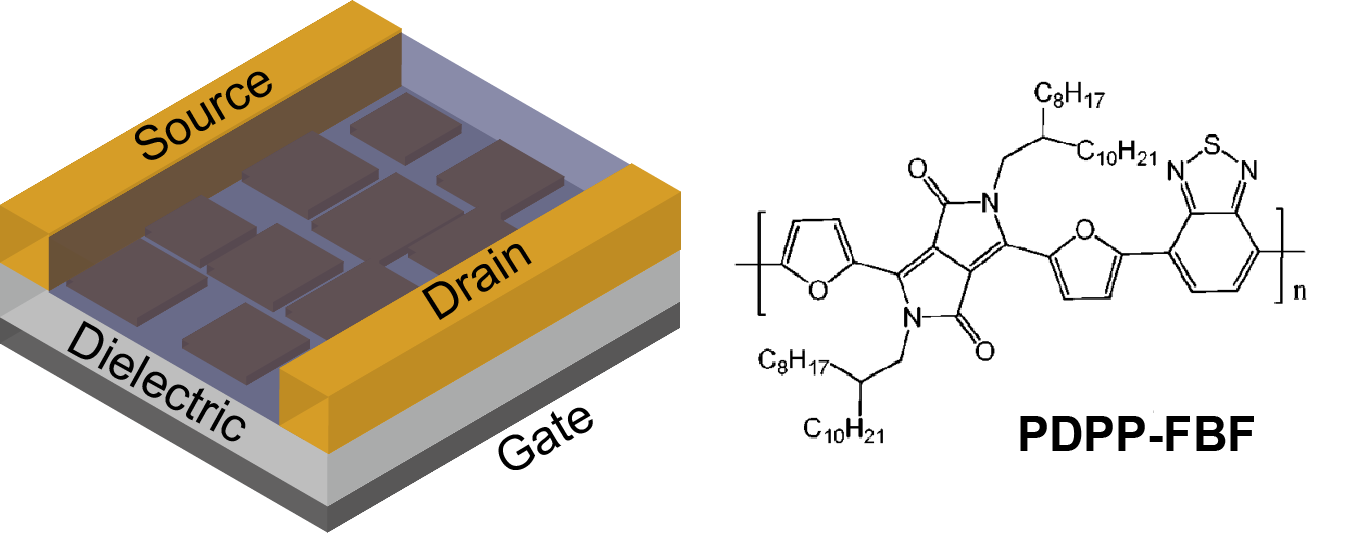


**Supplementary Figure 6.** Device structure of the hybrid transistor with PDPP-FBF and GO.


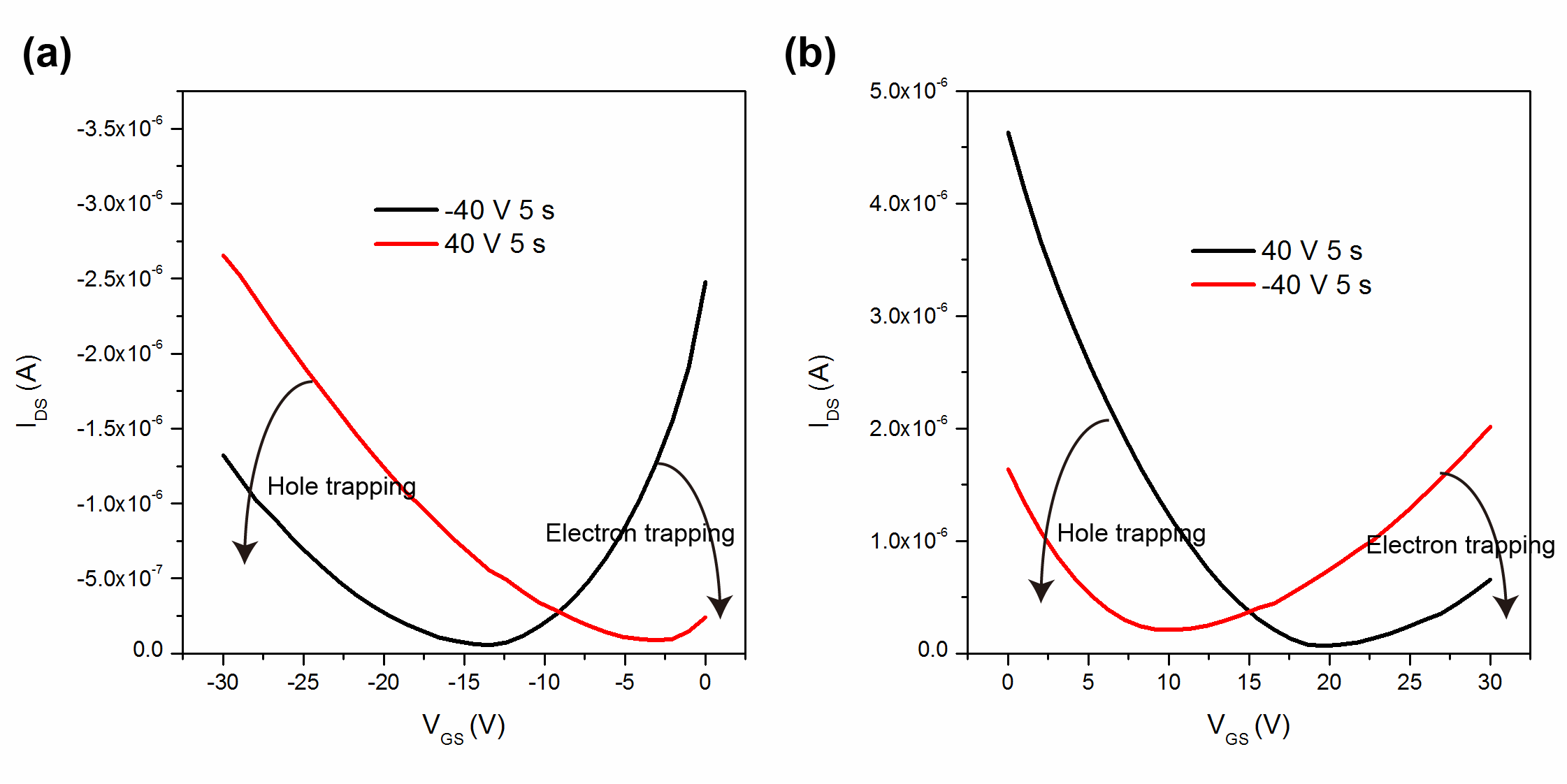


**Supplementary Figure 7.** (a) Transfer characteristics of the hybrid transistors based on PDPP-FBF at hole-enhancement mode. (b) Transfer characteristics of the hybrid transistors based on PDPP-FBF at electron-enhancement mode.


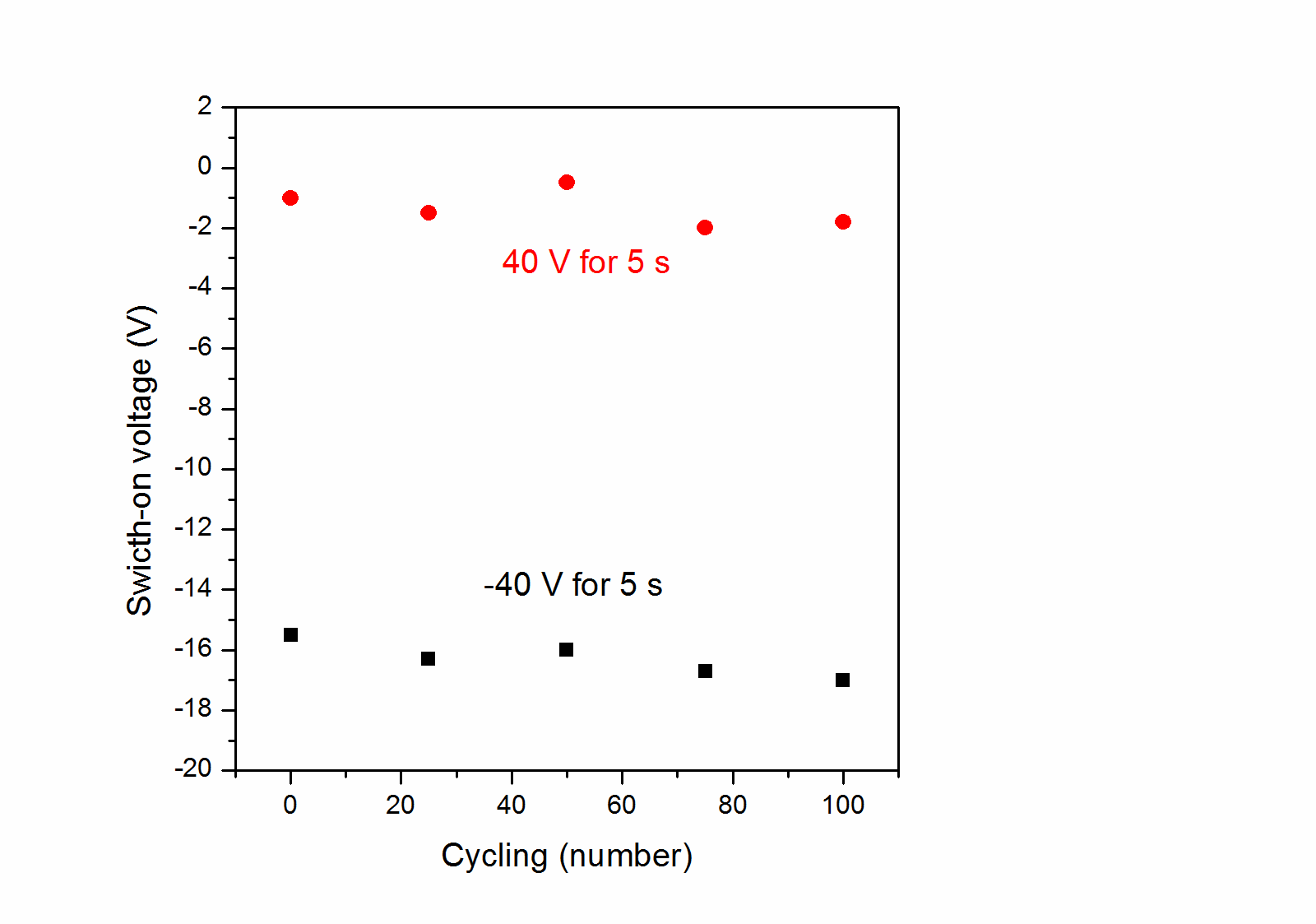


**Supplementary Figure 8.** Cycling test of the controlled polarity reversion.
